# Unsupervised Anomaly Detection for Intermittent Sequences Based on Multi-Granularity Abnormal Pattern Mining

**DOI:** 10.3390/e25010123

**Published:** 2023-01-07

**Authors:** Lilin Fan, Jiahu Zhang, Wentao Mao, Fukang Cao

**Affiliations:** College of Computer and Information Engineering, Henan Normal University, Xinxiang 453007, China

**Keywords:** anomaly detection, safety stock, intermittent sequence, unsupervised learning, after-sale parts management

## Abstract

In the actual maintenance of manufacturing enterprises, abnormal changes in after-sale parts demand data often make the inventory strategies unreasonable. Due to the intermittent and small-scale characteristics of demand sequences, it is difficult to accurately identify the anomalies in such sequences using current anomaly detection algorithms. To solve this problem, this paper proposes an unsupervised anomaly detection method for intermittent time series. First, a new abnormal fluctuation similarity matrix is built by calculating the squared coefficient of variation and the maximum information coefficient from the macroscopic granularity. The abnormal fluctuation sequence can then be adaptively screened by using agglomerative hierarchical clustering. Second, the demand change feature and interval feature of the abnormal sequence are constructed and fed into the support vector data description model to perform hypersphere training. Then, the unsupervised abnormal point location detection is realized at the micro-granularity level from the abnormal sequence. Comparative experiments are carried out on the actual demand data of after-sale parts of two large manufacturing enterprises. The results show that, compared with the current representative anomaly detection methods, the proposed approach can effectively identify the abnormal fluctuation position in the intermittent sequence of small samples, and also obtain better detection results.

## 1. Introduction

Aftermarket parts safety stock management and optimization are important tools for core manufacturing enterprises to meet the daily business needs of parts chain sites at all levels. A reasonable safety stock strategy can not only reduce the pressure of enterprise stocking and inventory cost, but also enhance the efficiency of enterprise inventory allocation and realize the safe operation of inventory [1]. In the actual business of enterprises, the safety stock strategy of after-sales parts is often determined by business needs such as replacement of defective parts and new project launches, but “abnormal parts demand” in non-business situations also occurs from time to time. For example, the accumulation of historical trouble work orders caused by a salesman’s error in reporting or natural factors such as seasonal hot weather, flurries, and sand can lead to abnormal demand. This kind of abnormal demand can easily interfere with the development of safety stock strategy and cause safety stock decision errors. Therefore, identifying abnormal demand and correcting it is an important problem that manufacturing companies need to solve in smart inventory management nowadays.

**Figure 1:**
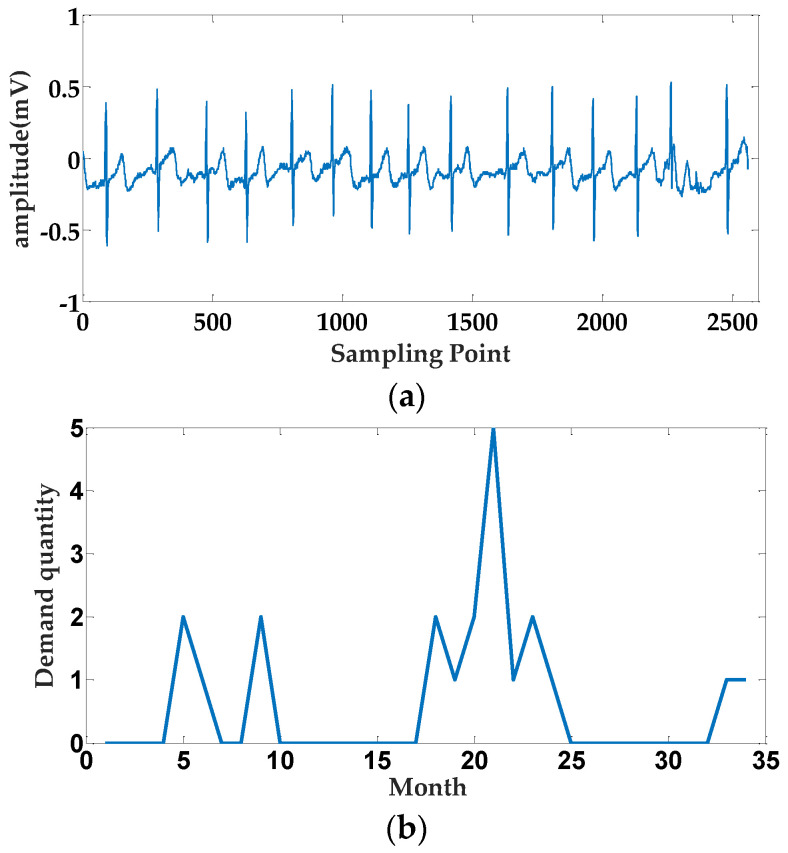
Comparison of different types of time series shapes. (**a**) Electrocardiogram timing data sequence of a patient. (**b**) A large vehicle manufacturer’s after-sales parts demand sequence.

Due to the uncertainty of aftermarket parts demand, resulting in poor continuity of parts data, weak time series, and more intervals between zero demand and demand, the aftermarket parts demand data present typical intermittent and small-sample characteristics compared with the general time series. As shown in Figure 1, Figure 1a gives the time series data of a patient’s electrocardiogram, which contains 2560 sampling points, and Figure 1b shows the 34-month demand plan data of a certain accessory for a large domestic manufacturing enterprise. In contrast, the sequence in Figure 1a has an obvious periodicity and trend, while the demand data sequence in Figure 1b is more sparse, and the regularity of data distribution is not obvious and has strong intermittency. For the abnormal demand detection of such intermittent sequences, enterprises mostly rely on experienced operation and maintenance experts to define them, which is slow and difficult to meet the demand for parts management of the large-scale business. Therefore, it is necessary to theoretically study the anomaly detection of intermittent time series to optimize the safety stock strategy and improve the operational efficiency of manufacturing enterprises.

Anomaly detection of time series is to detect a small number of points with outliers, oscillations and other anomalies in the time series data [2]. Due to the high cost of labeling time series data, which cannot be applied to large-scale data [3], currently, the most dominant method for time series anomaly detection is the unsupervised detection method. The most common unsupervised anomaly detection methods include One-Class Support Vector Machines (OCSVMs) [4], a Local Outlier Factor (LOF) [5], K-Nearest Neighbors (KNN) [6], and Isolation Forest (IForest) [7]. These models measure the outlier degree of data points and score anomalies by density, statistics, distance, and are computationally fast and suitable for small-sample data. With the rapid development of deep neural networks, deep learning has also been applied to time series anomaly detection [8]. Niu et al. proposed a hybrid LSTM-based VAE-GAN (Variational AudoEncoder-Generative adversarial networks) model for time series anomaly detection, which uses LSTM networks for training and detects anomalies based on reconstructed difference and discriminant results [9]. Ji et al. proposed the LSTMAD model to learn structural features from normal training data and then detect anomalies based on the prediction error of the observed data and using statistical strategies [10]. Such methods generally require a large amount of data for training. Although all the above methods have achieved better results, they are all based on the assumption of continuous data with more periodic changes for anomaly detection. Once the data have intermittent distribution, the above traditional detection methods based on density, statistics and distance are very easy to identify normal intermittent data as discrete points, while the depth methods are also difficult to learn the distribution characteristics of intermittent sequences, resulting in poor accuracy of anomaly detection. Since intermittent sequences are not only reflected in abnormal values at a certain time point but also have the phenomenon that the fluctuation pattern of the whole sequence shows abnormal posture, unlike the above-mentioned conventional time series abnormality detection practices, the abnormality detection of intermittent sequences needs to be combined with the characteristics of intermittent data to evaluate the abnormality in two dimensions—sequence and time point.

A comprehensive analysis shows that the key to improving the effect of intermittent time series anomaly detection is: (1) how to quantify the anomalous fluctuation pattern of intermittent sequences and identify the sequences with anomalous fluctuation posture; (2) how to effectively use the demand change characteristics and interval time information in intermittent sequences to improve the detection accuracy of small-sample intermittent sequences. To address the above two points, this paper proposes an unsupervised anomaly detection method for intermittent sequences based on multi-granularity anomaly pattern mining. First, a new anomaly fluctuation similarity index FlucSim (Fluctuation-Similarity) is constructed, which incorporates the squared coefficient of variation ($CV^{2}$) [11,12] difference between sequences and the maximal information coefficient (MIC) [13], and the identification of abnormal sequences is achieved by using cohesive hierarchical clustering. Secondly, in order to fully obtain the abnormal demand information in the abnormal sequences, this paper constructs demand variation features and interval features from the demand variation and demand interval characteristics of each time point in these sequences, and merges them into a Demand variation characteristics and interval characteristics (DVIC) matrix, and input to the support vector data description (SVDD) [14] model for hypersphere training and abnormal detection. The experimental results prove the effectiveness of the method in two large vehicle manufacturers’ aftermarket parts demand datasets.

The contributions of the work in this paper can be summarized as follows.

(1) An abnormal volatility metric for intermittent time series is proposed. The index not only considers the difference in volatility patterns between series but also the correlation between series, so as to achieve an the accurate quantification of abnormal volatility of intermittent time series.
(2) An unsupervised anomaly detection method for intermittent sequences based on multi-granularity anomaly pattern mining is constructed. Compared with the traditional anomaly detection methods, the method can identify anomalous sequences by mining the sequence anomaly fluctuation patterns from macroscopic and macroscopic perspectives, and effectively use the information of demand point value change in the sequence to locate the anomalous demand in anomalous sequences, which improves the intermittent sequence detection accuracy.

## 2. Related Theories

### 2.1. Demand Model Classification

In complex businesses such as heavy machinery manufacturing, automotive parts, and accessory after-sales maintenance services, and large equipment repair, the demand for spare parts presents different demand patterns. The current common classification scheme for demand is proposed by Syntetos and Boylan [15]. This scheme classifies demand sequences into four types: smooth, intermittent, unstable and lumpy based on the average demand interval (ADI) and the coefficient of variation of the squared demand size ($CV^{2}$) at the time of demand occurrence. ADI=N/Z, N denotes the length of the sequence, and Z denotes the number of non-zero demands in the sequence. $CV^{2}={\left(S/\bar{x}\right)}^{2}$. In the formula, S denotes the standard deviation of non-zero demands in the sequence, S is calculated as $S=\sqrt{\sum_{i=1}^{n}{\left(x_{i}-\bar{x}\right)}^{2}/n}$, $\bar{x}$ denotes the mean of non-zero demands, $x_{i}$ denotes the demand value of the i-th non-zero demand, and i=1,2,⋯,n, n denotes the number of non-zero demands. Since ADI and $CV^{2}$ can describe the characteristics of intermittent demand data very well [16], it is possible to classify the sequence of aftermarket parts demand in the actual business of the company.

### 2.2. Hierarchical Clustering

Hierarchical clustering is a kind of unsupervised learning clustering method, which can be divided into “bottom-up” cohesive hierarchical clustering or “top-down” split hierarchical clustering methods according to the direction of clustering. Both of clustering methods are stable [16]. However, cohesive hierarchical clustering starts with all the samples to be divided as initial clusters, i.e., each sample forms its own class, and then the clusters with high similarity are merged to form a larger cluster based on the predefined coalescence criterion, and iteratively merged upward until the set number of clusters, which has lower computational complexity. In contrast, split hierarchical clustering [17] is to initialize all samples to be divided into one class cluster, and then gradually split downward to form multiple smaller class clusters, and iteratively split to a set number of class clusters, which has a larger computational overhead. Therefore, cohesive hierarchical clustering is more widely used in practical application scenarios. Due to the existence of matrix computation in the hierarchical clustering algorithm, it has greater time and space complexity and is suitable for smaller datasets [16]. By defining the similarity index between sequences, hierarchical clustering is suitable for time series clustering [18]. At the same time, the clustering results can visually reflect the correlation degree between the sequences. So hierarchical clustering is chosen as the clustering method for intermittent time series.

### 2.3. SVDD

The support vector data description algorithm is a single-value classification algorithm whose main function is to be able to distinguish between target and non-target samples. The main process of this algorithm is as follows: firstly, the data are mapped from the original space to a higher latitude feature space, and then a hypersphere with the smallest volume is found in this feature space, and all target samples are included in the hypersphere as much as possible while considering outliers. To construct this minimum hypersphere, the support vector data description algorithm needs to optimize the following objective function.

(1) $$\left\{ \begin{array}{l} \underset{a,R,\xi }{\min}\;R^{2}+C\sum_{i=1}^{n}\xi_{i}\\ s.t.\;{\left\|\Phi (X_{i})-a\right\|}^{2}\leq R^{2}+\xi_{i},\\ \;\xi_{i}\geq 0,\forall i=1,2,\cdots ,n \end{array}\right.$$

where a is the center of the hypersphere, R is the radius of the hypersphere, ξ is the relaxation factor, C is the penalty factor that weighs the volume of the hypersphere and the misspecification rate, $X_{i}(i=1,2,\cdots ,n)$ is the training sample, n is the number of samples, and $\Phi \left(X_{i}\right)$ is the function that maps the training sample $X_{i}$ to the high-dimensional space. The detailed derivation process can be found in the literature [14].

## 3. Unsupervised Anomaly Detection Method for Intermittent Sequences Based on Multi-Granularity Anomaly Pattern Mining

This section proposes an unsupervised anomaly detection method for intermittent time series, which consists of two main parts: the first part is to find the patterns of anomalous fluctuations in intermittent time series and identify the anomalous sequences, and the second part is to perform unsupervised anomaly detection on these sequences.

In the first part, the information on the regularity of multiple dimensions of the sequences is taken into account, and this paper proposes a new anomalous fluctuation similarity index and constructs a corresponding cohesive hierarchical clustering algorithm for clustering. The role of this part is to identify anomalous sequences with abnormal fluctuations from known sequences.

**Figure 2:**
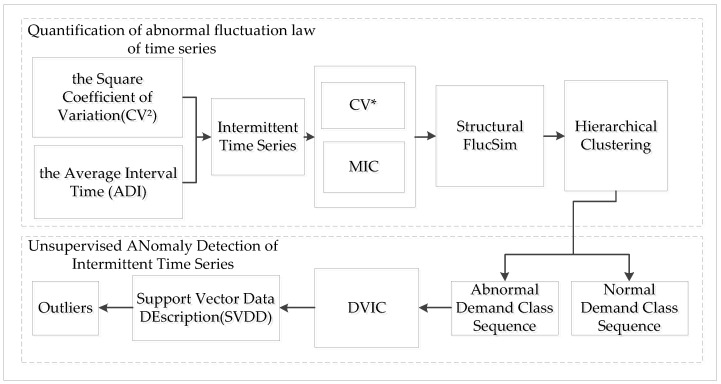
Flow chart of the method.

In the second part, using the information on the number of demands and non-zero demand interval time at each time point in the anomalous sequence, this paper reorganizes a sequence into a matrix of demands at a set of time points and inputs this matrix into the SVDD model for unsupervised anomaly detection. The function of the second part is to improve the unsupervised anomaly detection of intermittent sequences with small samples by using the variation of demands in the sequence and the intermittency of the sequence. The flow chart of the method in this paper is shown in Figure 2. The details are described below.

### 3.1. Construction of Anomalous Fluctuation Similarity Index for Intermittent Series

**Figure 3:**
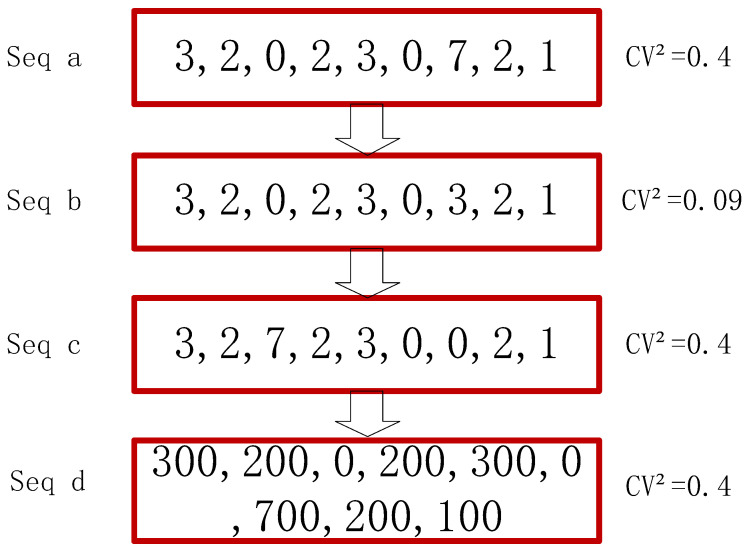
Schematic diagram of *CV*^2^ calculation for intermittent time series.

In the intermittent time series characterization, $CV^{2}$ indicates the squared coefficient of variation, which reflects the stability of the series and can effectively describe the fluctuating changes in the demand of the series. Some examples are given here to illustrate, as shown in Figure 3: For four intermittent sequences a, b, c, and d, the four sequences have the same sparsity and the calculated ADI is the same, so the size difference of $CV^{2}$ of the sequences can be directly compared. For the two sequences a and b in example 1, the basic elements and the number of elements in the two sequences are the same, but the element value of sequence a at position 7 is “7”, while the value of the element in the same position of sequence b is “3”, the volatility of sequence a is greater, so the $CV^{2}$ of sequence a is larger than that of sequence b, indicating that the size of the element has a greater impact on $CV^{2}$. In example 2, the sequences a and c only change the position of element “7”, but the overall volatility of the sequences does not changed, and the $CV^{2}$ of sequence a and c does not change, indicating that the change in the position of the elements does not affect the $CV^{2}$. Similarly, from the above two conclusions, the reason for the difference in $CV^{2}$ of sequences b and c can be known. In example 3, sequences a and d, sequence d changes in quantity level based on a, but does not affect the $CV^{2}$ value, it can be concluded that $CV^{2}$ is only concerned with the fluctuation of the sequence and can measure the fluctuation of demand for different kinds of accessories.

However, $CV^{2}$ can only describe the fluctuating evolution information of a single sequence, which is not sufficient to reflect the similarity between different series. In order to measure the volatility correlation between different intermittent series, this subsection introduces the MIC indicator, which can effectively detect linear and nonlinear correlations between variables, as well as measure the evolutionary trend of a series, and the specific calculation method is described in the literature [19]. Therefore, the combination of $CV^{2}$ and MIC indicators can effectively measure the evolutionary trend of series fluctuations.

Based on the above analysis, this section designs the serial volatility difference indicator $CV^{*}$ to measure the volatility difference between the two series as follows.

(2) $$CV^{*}\left(T_{i},T_{j}\right)=\frac{1}{1+e^{-k\left|CV^{2}(T_{i})-CV^{2}(T_{j})\right|}}$$

where $CV^{2}\left(T_{i}\right)$ and $CV^{2}(T_{j})$ denote the squared coefficients of variation of the sequences $T_{i}$ and $T_{j}$, respectively, and k is the variable coefficient. It can be seen that the smaller the difference in the values of $CV^{2}$ between the two sequences, the smaller the values of $CV^{*}$ between these two sequences, i.e., the smaller the difference in the fluctuations of the two sequences.

In order to measure the similarity of fluctuations between sequences, based on the $CV^{*}$ indicator and combined with the *MIC* indicator, this paper constructs an abnormal fluctuation similarity indicator FlucSim. For a given set of sequences $T=\left\{T_{1},T_{2}\cdots ,T_{n}\right\}$, n is the number of sequences, where the first i sequence $T_{i}=\left\{x_{1},x_{2},\cdots ,x_{m}\right\}$, m is the number of elements in the sequence, and then FlucSim indicator is shown in Equation (3).

(3) $$\begin{array}{cc} FlucSim\left(T_{i},T_{j}\right)= & \left(1-MIC\left(T_{i},T_{j}\right)\right)⊙\\ & CV^{*}\left(T_{i},T_{j}\right) \end{array}$$

where $\mathrm{MIC}\left(T_{i}{,T}_{j}\right)$ denotes the *MIC* value between sequences $T_{i}$ and sequences $T_{j}$, ⊙ denotes Hadamard product notation. Hadamard product is a class of matrix operations in which two matrices of the same dimension are multiplied by the corresponding position elements and produce a third matrix of the same dimension. ${\mathrm{CV}}^{*}\left(T_{i}{,T}_{j}\right)$ indicates the fluctuation difference between sequences $T_{i}$ and sequences $T_{j}$. As can be seen from Equation (3), the formula integrates the fluctuation difference and correlation information between intermittent sequences. The smaller the FlucSim, the more similar the fluctuation pattern and sequence trend between two sequences, and the more detectable information it contains.

In order to fully mine the sequence anomalous patterns, firstly, using the similarity index of anomalous fluctuations calculated by Equation (3), this section constructs a coarse-grained anomalous pattern mining hierarchical clustering algorithm for intermittent time series, which divides intermittent sequences containing anomalous sequences into normal and anomalous sequences from the bottom up. The steps are as follows.

(1) Partitioning the original dataset based on the ADI and $CV^{2}$ indicators to identify intermittent sequences.
(2) Considering each intermittent sequence as a class cluster.
(3) Calculating the similarity distance between each class cluster using Equation (4).
  (4) $$D_{A,B}=1/\left(\left|A\right|\times \left|B\right|\right)\sum_{T_{i}\in A}\sum_{T_{j}\in B}FlucSim(T_{i},T_{j})$$
  where 1≤i,j≤n,i≠j, |A| and |B| denote the number of sequences contained in the class cluster A and the class cluster B that participate in the distance calculation, respectively, and n denotes the number of sequences.
(4) Merging the two closest class clusters into one class cluster.
(5) Repeating steps (3) (4) until all sample sequences are divided into a set number of classes.
(6) Calculating the $CV^{2}$ of each sequence in the class cluster obtained from step (5), and calculate the average $CV^{2}$ value in each class cluster, with the $CV^{2}$ value exceeding a preset threshold set as an abnormal sequence, and the rest are normal sequences.

### 3.2. Unsupervised Anomaly Detection Method for Intermittent Sequences Based on Multi-Granularity Anomaly Pattern Mining

In the actual business of an enterprise, the sudden change in after-sales parts demand and the length of interval between adjacent demands may often be two important factors that imply abnormal parts demand. Therefore, in order to fine-grained mining and analyze the abnormal demand point characteristics in the abnormal sequence, this section constructs demand change features and interval features based on each time point demand in the abnormal sequence and merges them into a matrix of DVIC to describe the demand change and demand interval of the time point demand by doing a feature extraction for each time point demand in the sequence and discretizing the sequence into a set of features of the time point demand. For the set of anomalous sequences $T=\left\{T_{1}{,T}_{2},\cdots ,T_{M}\right\}$, M denotes the number of anomalous sequences, and the specific approach to construct features for the i-th anomalous sequence $T_{i}=\left\{x_{1}{,x}_{2},\cdots {,x}_{m}\right\}$ is as follows.

(1) Demand change features: the three-dimensional demand change characteristics are obtained by calculating the demand difference between the current time node demand $x_{t}$ and the previous time node demand $x_{t-1}$, the first two-time node demands $x_{t-2}$ and the first three time node demands $x_{t-3}$, which reflect the demand change.
(2) Demand interval features: The length of the demand interval is reflected by calculating the interval between the demand at the non-zero demand time node $x_{i}$ and the demand at the previous non-zero demand time node $x_{j}$, where 1≤t,i,j≤m and m represent the length of the sequence $T_{i}$. DVIC The matrix is shown in Table 1.

Then, the matrix of DVIC constructed from the anomalous sequence $T_{i}$ is divided into training data and test data according to a certain ratio, and the default training data are mostly normal demand, which is input to the SVDD model for hypersphere training. The trained hyperspheres are used to check the occurrence position of outliers in the test data. Unsupervised anomaly detection of intermittent sequences is achieved in this way. Similarly, the same approach is used for the detection of other anomalous sequences.

Combining the above steps, this chapter constructs an unsupervised anomaly detection method for intermittent sequences based on multi-granularity anomaly pattern mining. The steps of the method are as follows.

Input: Intermittent time series $T=\left\{T_{1}{,T}_{2},\cdots {,T}_{n}\right\}$, where $T_{i}=\left\{x_{1}{,x}_{2},\cdots {,x}_{m}\right\}$, number of class clusters K.

Step 1: Construct coarse-grained anomaly pattern mining hierarchical clustering algorithm to realize sequence clustering.

(1) Set $T_{i}$ into a class $c_{i}$ separately and obtain the class cluster.
  $$C=\left\{c_{1},c_{2},\cdots ,c_{n}\right\}$$
(2) Find the two class clusters $c_{i}$ and $c_{j}$ with the shortest similarity distance from the class cluster C. The formula for calculating the similarity distance is shown below.
  (5) $$D\left(c_{i},c_{j}\right)=\frac{1}{\left|c_{i}\right|\times \left|c_{j}\right|}\times \sum_{T_{i}\in c_{i}}\sum_{T_{j}\in c_{j}}FlucSim\left(T_{i},T_{j}\right)$$
(3) Merge the class clusters $c_{i}$ and $c_{j}$ into a new class cluster $c_{n}$ and update the class cluster C.
(4) If the class cluster C has been divided into K class clusters, go to step (5); otherwise loop to step (2).
(5) Calculate the $CV^{2}$ for each sequence in C separately and calculate the average $CV^{2}$ for all sequences in each category.
(6) If the average $CV^{2}$ is smaller than the preset threshold, the corresponding class is set to the normal class normal_class, and the rest to the abnormal class:
  $$\mathrm{abnormal}\text{\_}\mathrm{class}=\left\{c_{1},c_{2},\cdots ,c_{p}\right\},\;p+1=K.$$

Step 2: For each sequence in abnormal_class, construct point-in-time demand change features and interval features and merge them into a DVIC matrix, and then construct SVDD anomaly detection models for unsupervised anomaly detection separately.

Output: The abnormal values in the test data for each abnormal sequence in abnormal_class.

## 4. Experimental Analysis

### 4.1. Dataset Introduction

**Figure 4:**
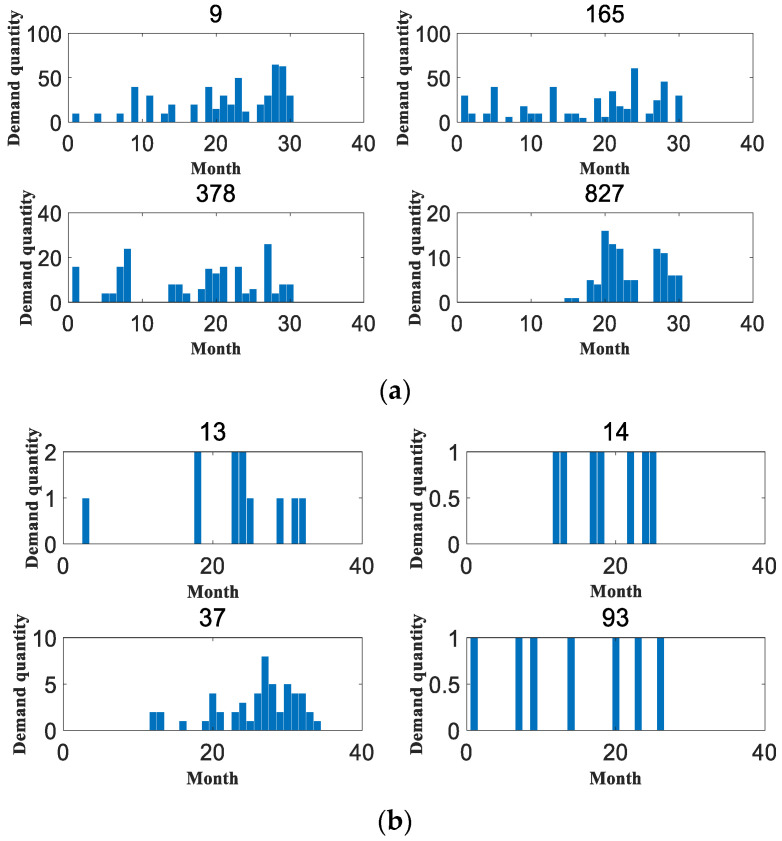
The original distribution of the experimental data in this paper. (**a**) Distribution of original data in dataset 1. (**b**) Distribution of original data in dataset 2.

**Figure 5:**
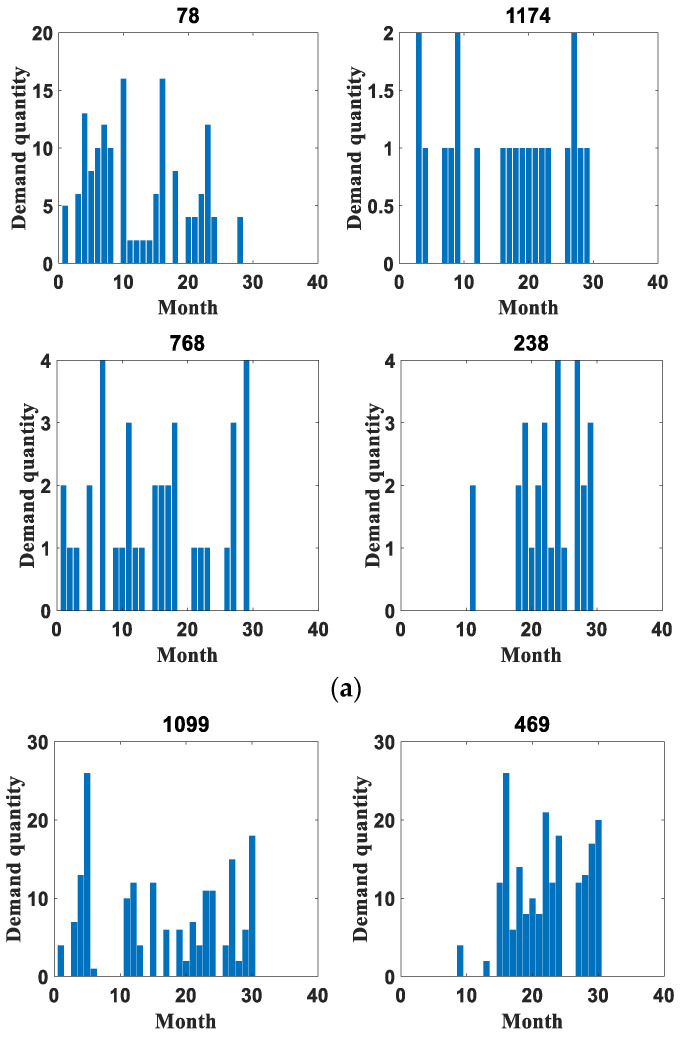

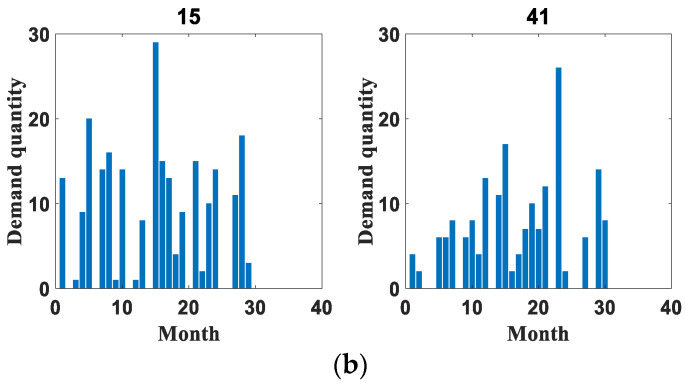
Example of partial clustering results for dataset 1. (**a**) Example of distribution of partial normal sequences. (**b**) Example of distribution of some anomalous sequences.

**Figure 6:**
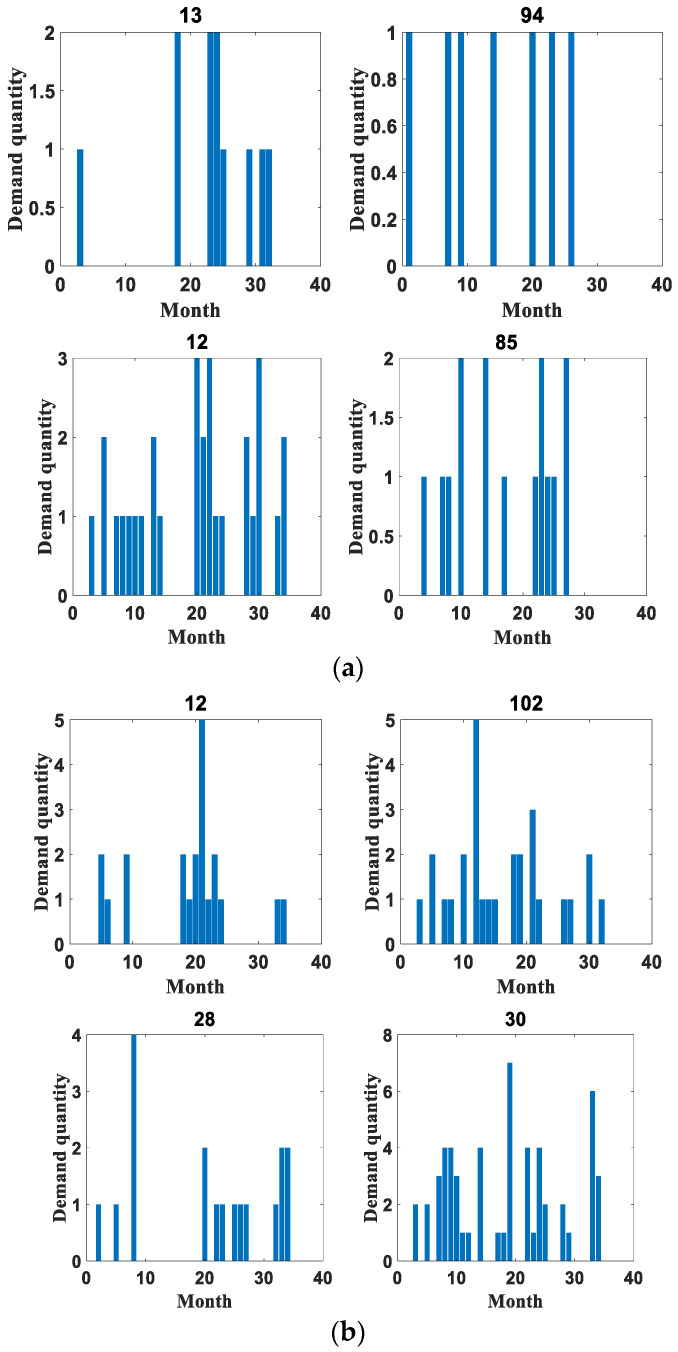
Example of partial clustering results for dataset 2. (**a**) Example of distribution of partial normal sequences. (**b**) Example of distribution of some anomalous sequences.

**Figure 7:**
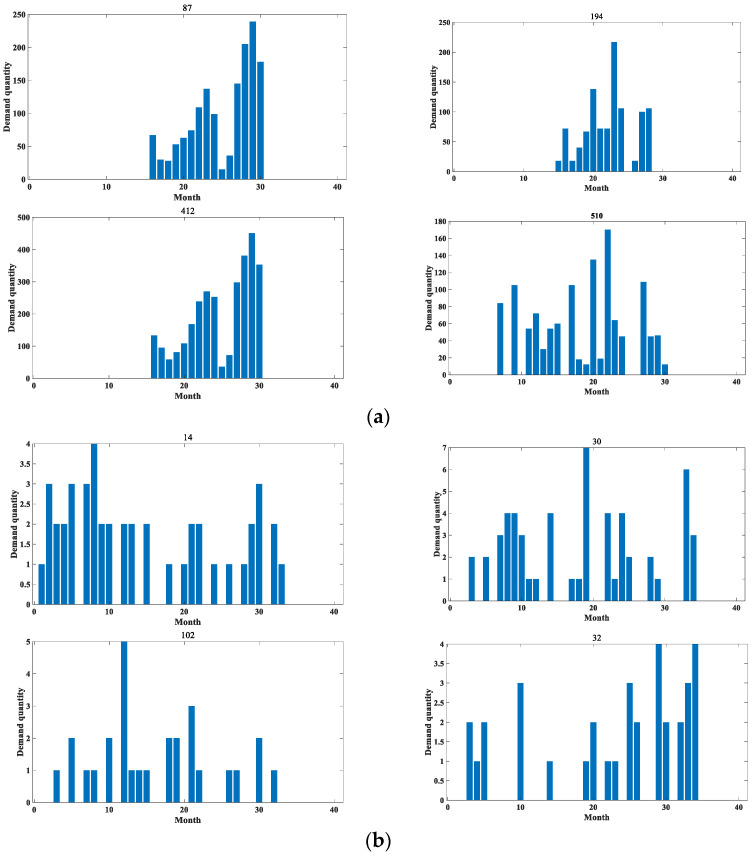
Some examples of abnormal sequences obtained using DTW clustering. (**a**) Example of anomalous sequence using DTW clustering for dataset1. (**b**) Example of anomalous sequence using DTW clustering for dataset2.

**Figure 8:**
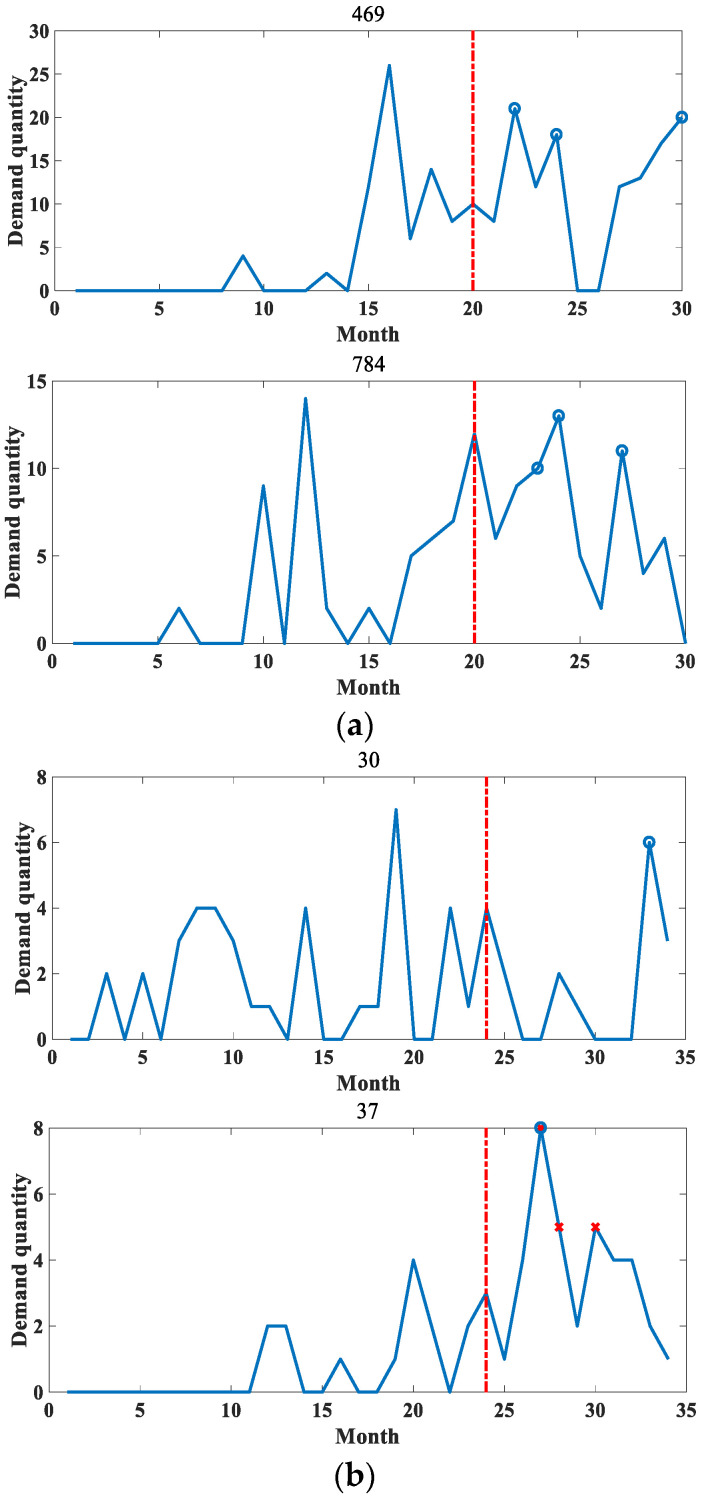
An example diagram of anomaly detection results before feature construction. (**a**) Example of anomaly detection results for part of dataset 1. (**b**) Example of anomaly detection results for part of dataset 2.

**Figure 9:**
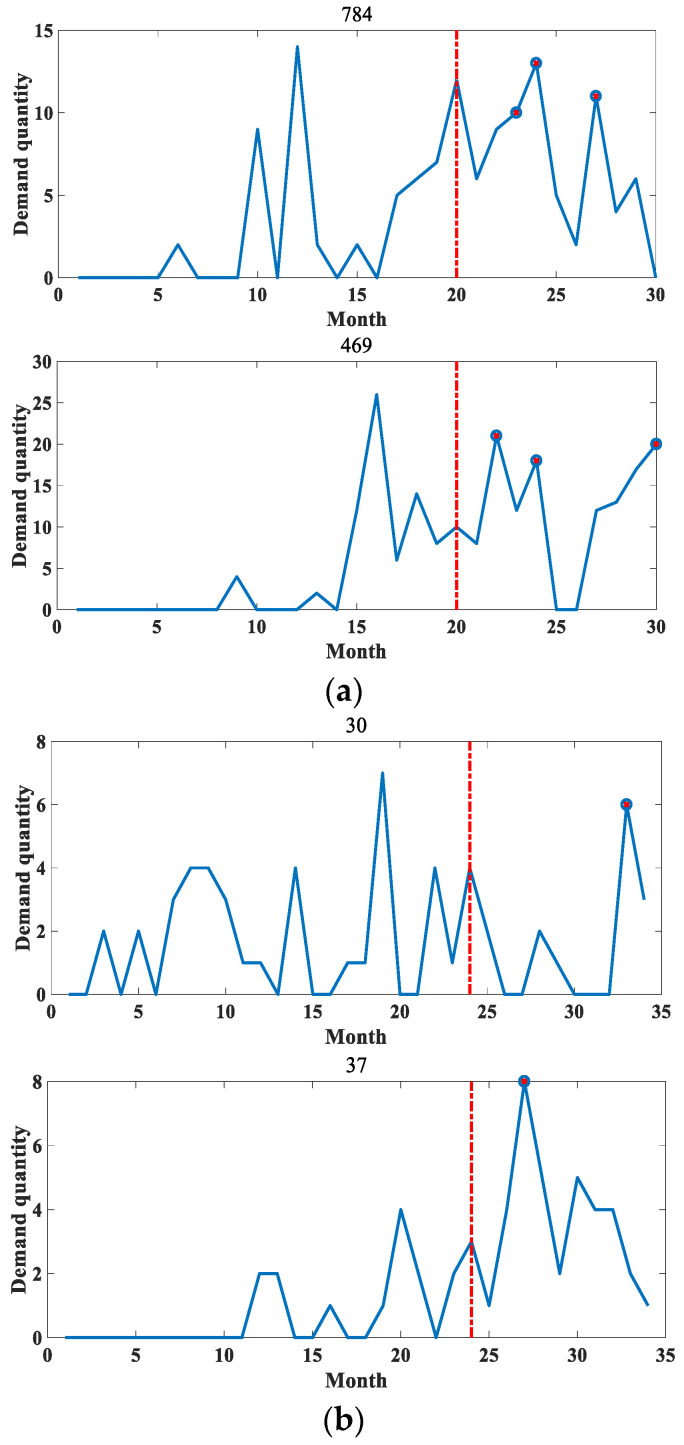
An example of anomaly detection results for some anomalous sequences of the method in this paper. (**a**) Example of anomaly detection results for some parts of dataset 1. (**b**) Example of anomaly detection results for some parts of dataset 2.

In order to verify the reliability of this paper, the application effect is verified on two datasets, respectively. The first dataset is the spare parts demand of heavy equipment enterprises, which was provided by Zoomlion China, a famous vehicle manufacturer, in the Fifth National Industrial Internet Data Innovation and Application Competition. The access link is “https://www.industrial-bigdata.com/Competition” (accessed on 4 January 2023). After registration, users can download the dataset for test. The second dataset is an after-sales parts demand dataset of a large vehicle manufacturing enterprise provided by a high-speed train manufacturing company in China with which this paper has cooperation. This company is a leading global supplier of rail transportation equipment in terms of size, variety and technology. We feel very sorry that we cannot publish the dataset to the public due to a data privacy agreement with the provider. Among them, the heavy equipment enterprise spare parts demand dataset provides 1200 types of spare parts with 30 months of historical data from January 2018 to June 2020. The dataset mainly contains the historical sales data of aftermarket spare parts, the retention of equipment corresponding to the spare parts and the start-up of equipment corresponding to the spare parts in this period. It is denoted as dataset 1 in the rest of the content. The aftermarket parts demand dataset of a large vehicle manufacturer contains a total of 16 warehouses, with 23 material replacement groups for a total of 34 months from November 2018 to August 2021. This dataset is represented in the remaining content as dataset 2. Both datasets are identified by month to determine the time nodes, and their specific details are shown in Table 2. In addition, Table 3 shows some statistical indicators of the two datasets, including the mean and standard deviation. The original datasets are huge, with hundreds of sequences each. In terms of the *ADI* and *CV²* indicators, some sequences with obvious intermittent characteristics and large data fluctuations are selected for comparative evaluation, as shown in Figure 4, Figure 5, Figure 6, Figure 7, Figure 8 and Figure 9. Other sequences in the dataset have similar effects. The above two datasets have been uploaded to “https://github.com/Miss-Lyan/z_datasets-” (accessed on 4 January 2023).

Here, Table 3 gives the statistical count information for both datasets.

Figure 4 shows some of the accessory data in the two datasets. It can be seen that the demand sequence of accessories has a obvious intermittency and a certain distribution similarity between different sequences. In order to improve the drawing efficiency, the figure titles of 0 and the following effect figures indicate the numbers of accessories.

### 4.2. Evaluation Metrics

As with most anomaly detection problems, this paper uses precision, recall and *F*1 score as evaluation metrics for this paper’s method and comparison methods. Their calculation formulas are shown below.

(6) $$Precision=\frac{TP}{TP+FP}$$

(7) $$Recall=\frac{TP}{TP+FN}$$

(8) $$F1=2*\frac{Precision*Recall}{Precision+Recall}$$

### 4.3. Experimental Results

The coarse-grained anomaly pattern mining hierarchical clustering algorithm in Section 3.1 was used to cluster the two datasets separately. The algorithm clustered the datasets into different types of sequences, which are normal sequences and anomalous sequences. The clustering results are shown in Figure 5 and Figure 6.

It can be seen that the values in the abnormal sequences are highly random and volatile, with demand at certain points in time being significantly greater than the general level of demand. This sudden demand is bound to have a special impact on the present material inventory management. Therefore, it has detectability. In contrast, the normal sequences have small demand, relatively stable overall demand and have a certain distribution pattern, so there is no need for anomaly detection for such.

In order to verify the superiority of the anomalous fluctuation similarity index Flusim proposed in this paper, this paper adds the use of Dynamic Time Warping algorithm (DTW) [19] as an index for screening anomalous sequences based on the original experiment. The DTW algorithm is used to calculate the similarity between two time series sex by extending and shortening the time series. This algorithm is currently a more used method to calculate the similarity between sequences.

As shown in Figure 7, the DTW indicator will be influenced by its own characteristics when performing sequence screening. It will group sequences with similar patterns into one category. However, the graph of similar morphology may often indicate that the accessories that match these similar shapes are sets of outgoing libraries that do not reflect the abnormal fluctuations of the sequences. Therefore, using the DTW indicator to screen abnormal sequences does not achieve good results.

Based on the sequence clustering, the *DVIC* matrices of the obtained anomalous sequences for the point-in-time demands are constructed separately, and the unsupervised anomaly detection modeling is performed using SVDD. Considering that the sequence lengths in both datasets are relatively short, the first 20 months of demand data of the anomaly sequence in dataset 1 are used as training data to test the anomalies in the last 10 months in this experiment. The demand data of the first 24 months of the anomalous sequence of dataset 2 are used as training data to examine the anomalies of the last 10 months. Among them, the real labels of the test data are obtained through communication with relevant technical experts of the enterprises.

In order to verify the reasonableness of the sequence DVIC matrix being constructed, this paper conducted experimental comparison before and after feature generation on two datasets, respectively, and the comparison results are shown in 0 and 0. In these figures, the red dashed line divides a sequence into two parts, the former part represents the training data of the model and the latter part represents the test data. The anomaly detection results of the sequences are also labeled on the test set, with the symbol “o” marking the true label of the sequence and the symbol “x” indicating the anomaly detection results.

The comparison shows that the anomaly detection result is better after feature construction. This is mainly because when SVDD modeling is performed on the original data, the model is more influenced by the training data when training the hypersphere, and the outliers in the training data will cause the radius of the hypersphere to increase [20,21]. Considering only the outliers of the numerical dimension in the sequence, all the outlier demands cannot be effectively detected. Additionally, after feature construction, the model not only takes into account the influence factors of outliers during training, but also is able to detect demand data with obvious demand changes and abnormal intervals. The model is less influenced by a single dimension, so the anomaly detection effect is improved, which verifies the effectiveness of the method in this paper.

### 4.4. Comparison Method

The comparison methods contain both traditional unsupervised anomaly detection methods such as OCSVMs and the latest unsupervised anomaly detection method COPOD [22]. The details are shown in Table 4.

**Figure 10:**
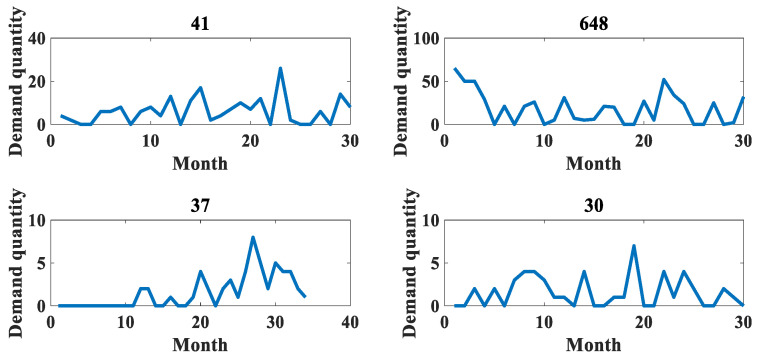
Sequence demand chart of experimental accessories.

Since the abnormal sequences account for a relatively small proportion of all parts demand sequences, in dataset 1, the sequence of abnormal accessories 41 and 648 is selected as the experimental object in this paper. In dataset 2, the sequence of accessories 37 and 30 is selected as the experimental object. The sequence demand trend of these accessories is shown in Figure 10.

Table 5 and Table 6 list the detection results and performance metrics of this paper’s method and other anomaly detection benchmark methods on two experimental datasets of anomalous sequences. In the column of time node detection results in the table, the symbol “-” indicates that the detected demand is a normal point, and the symbol “o” indicates an abnormal point.

The accessory anomaly demand sequences used in the experiments are obtained by the processing method in this paper. Due to the small number of anomalous sequences, this paper takes out two kinds of accessories in two datasets for experiments, respectively. The comparison method directly performs unsupervised anomaly detection on the unprocessed anomaly sequences.

As can be seen from the two tables above, the detection results of the OCSVM algorithm on several accessory demand data perform poorly relative to other methods, This is because it is difficult to train a suitable support vector to classify demand samples from a short length and large fluctuation. The IForest algorithm is an integrated method based on data cutting, which can determine the degree of abnormality based on the number of times the sample points are split apart as needed. It can be inferred from the result table that the method can detect some abnormal demands with few cuts, but it also mistakenly detects some normal demand points as abnormal demands. The method does not have high accuracy in detecting intermittent data. The The KNN algorithm performs anomaly detection based on the class of samples in the majority of the K most adjacent samples in the feature space. It can be seen that the method has high detection accuracy in detecting anomalous samples with prominent local demand changes, but the detection results on continuous, demand-intensive and stable demand data have higher errors. The LOF algorithm marks sample points located in sparse regions as anomalous by searching the nearest neighbors by means of density estimation. Similar to the KNN algorithm, it is also better at detecting local anomalous salient demands. The COPOD algorithm evaluates anomalies by estimating the probability of the tail end of sample points, in other words the probability that the sample points are distributed at extreme locations. The method has good detection results on overall demand data, but for demand-type data with growing demand, the method flags some new demand samples with large values as anomalous demand as the number of samples increases.

It can be seen that the traditional method of anomaly detection by density and distance is not good at detecting anomalous demands in demand sequences because it ignores the changing situation of demands in the sequences and the intermittency of the sequences. The method in this paper is to improve the detection accuracy by constructing DVIC matrix for the original sequence, fine-grained analysis of each demand characteristic in the sequence and the interval characteristics of non-zero demand, and mining the abnormal information in the demand sequence from multiple dimensions. It can be seen that in most cases, this method can obtain better detection results and is more stable than other methods. The effectiveness of this method is verified by the fact that the *F*1 scores of this method are higher than or equal to those of other detection methods in two different datasets.

## 5. Concluding Remarks

This paper studys an unsupervised anomaly detection method for intermittent sequences based on multi-granularity anomaly pattern mining. The method can effectively identify coarse-grained anomalous patterns in known sequences and screen out anomalous sequences in terms of volatility differences in intermittent time series and evolutionary similarity information between sequences. The DVIC matrix of time point demand is also constructed using the demand size of each time point of the anomalous sequence for unsupervised anomaly detection modeling. The advantage of this method is that it can effectively utilize the variation of demand in the sequence and the intermittency of the sequence to improve the detection accuracy of intermittent time sequences under small samples. The experimental results show that the method in this paper can effectively detect anomalous demand points with different intermittency distribution characteristics and has good practicality.

In the next step, we plan to combine the abnormal demand points of intermittent time series with the enterprise inventory stocking plan. We will classify different types of abnormal demand and construct fine-grained inventory optimization strategies for different types of abnormal categories to meet the actual inventory stocking demand of enterprises and reduce their inventory stocking pressure.

## Figures and Tables

**Table 1:** *DVIC* matrix.

Original Sequence	Demand Change Characteristics			Demand Interval Characteristics
	Previous Demand Difference	Top Two Demand Differentials	Top Three Demand Differentials	
0	0	0	0	0
1	1	0	0	2
0	−1	0	0	0
1	1	0	1	2
0	−1	0	−1	0
0	0	−1	0	0
0	0	0	−1	0
0	0	0	0	0
1	1	1	1	5
0	−1	0	0	0
0	0	−1	0	0
0	0	0	−1	0
0	0	0	0	0
0	0	0	0	0
0	0	0	0	0

**Table 2:** Experimental dataset information.

Dataset	Number of Samples	Number of Features	Properties
Heavy equipment parts requirements dataset	1200	8	Material number, sales quantity, sales time, number of hosts kept, host type, hours started in the week, number of hosts started in the week, host type
A large vehicle manufacturer’s after-sales parts demand dataset	684	4	Warehouse number, part number, item number, month, monthly demand

**Table 3:** Dataset Statistical Indicators.

Dataset		Mean	Standard Deviation
Heavy equipment parts requirements dataset	Min	0.4988	0.6515
	Mode	1.007	1.2143
	Median	1.669	1.7983
	Max	93.7312	132.0505
A large vehicle manufacturer’s after-sales parts demand dataset	Min	0.324	0.648
	Mode	0.5277	1.4133
	Median	0.4842	1.1151
	Max	1.6129	5.595

**Table 4:** Comparison methods.

Algorithm Type	Algorithm Name
Linear model	OCSVM [4]
Based on density	LOF [5]
Based on distance	KNN [6]
Based on statistics	COPOD [22]
Tree based	IForest [7]

**Table 5:** Table of experimental comparison results for dataset 1.

Accessory Number	Algorithm Name	Time Node Detection Results										Performance Index %		
		21	22	23	24	25	26	27	28	29	30	Precision	Recall	*F*1
41	Real Tags	-	-	o	-	-	-	-	-	o	-			
	OCSVM	o	-	o	-	-	-	-	-	o	-	100.00	87.50%	93.33
	IForest	o	-	o	-	-	-	-	-	o	-	100.00	87.50	93.33
	KNN	-	-	o	-	-	-	-	-	o	-	100.00	100.00	100.00
	LOF	-	-	o	-	-	-	-	-	-	-	88.89	100.00	94.12
	COPOD	-	-	o	-	-	-	-	-	o	-	100.00	100.00	100.00
	Methodology of this article	-	-	o	-	-	-	-	-	o	-	100.00	100.00	100.00
648	Real Tags	-	o	-	-	-	-	-	-	-	-			
	OCSVM	-	o	o	o	-	-	o	-	o	o	100.00	44.44	61.54
	IForest	-	o	-	-	-	-	-	-	-	-	100.00	100.00	100.00
	KNN	-	-	-	-	-	-	-	-	-	-	90.00	100.00	94.74
	LOF	-	-	-	-	-	-	-	-	-	-	90.00	100.00	94.74
	COPOD	-	o	-	-	-	-	-	-	-	-	100.00	100.00	100.00
	Methodology of this article	-	o	-	-	-	-	-	-	-	-	100.00	100.00	100.00

The symbol “-” indicates that the detected demand is a normal point, and the symbol “o” indicates an abnormal point.

**Table 6:** Table of experimental comparison results for dataset 2.

Accessory Number	Algorithm Name	Time Node Detection Results										Performance Index %		
		25	26	27	28	29	30	31	32	33	34	Precision	Recall	*F*1
37	Real Tags	-	-	o	-	-	-	-	-	-	-			
	OCSVM	-	o	o	o	-	o	o	o	-	-	100.00	44.44	61.54
	IForest	-	o	o	o	-	o	o	o	-	-	100.00	44.44	61.54
	KNN	-	o	o	o	-	o	o	o	-	-	100.00	44.44	61.54
	LOF	-	o	o	o	-	o	o	o	-	-	100.00	44.44	61.54
	COPOD	-	o	o	o	-	o	o	o	-	-	100.00	44.44	61.54
	Methodology of this article	-	-	o	-	-	-	-	-	-	-	100.00	100.00	100.00
30	Real Tags	-	-	-	-	-	-	-	-	o	-			
	OCSVM	o	-	-	o	-	-	-	-	o	-	100.00	77.78	87.50
	IForest	o	-	-	o	-	-	-	-	o	-	100.00	77.78	87.50
	KNN	-	-	-	-	-	-	-	-	o	-	100.00	100.00	100.00
	LOF	-	-	-	-	-	-	-	-	o	-	100.00	100.00	100.00
	COPOD	-	-	-	-	-	-	-	-	o	-	100.00	100.00	100.00
	Methodology of this article	-	-	-	-	-	-	-	-	o	-	100.00	100.00	100.00

## Data Availability

Not applicable.
